# The Combination of Molecular Hydrogen and Heme Oxygenase 1 Effectively Inhibits Neuropathy Caused by Paclitaxel in Mice

**DOI:** 10.3390/antiox13070856

**Published:** 2024-07-17

**Authors:** Ignacio Martínez-Martel, Xue Bai, Rebecca Kordikowski, Christie R. A. Leite-Panissi, Olga Pol

**Affiliations:** 1 Grup de Neurofarmacologia Molecular, Institut de Recerca Sant Pau, 08041 Barcelona, Spain; 2 Grup de Neurofarmacologia Molecular, Institut de Neurociències, Universitat Autònoma de Barcelona, 08193 Barcelona, Spain; 3 Department of Psychology, Faculty of Philosophy Science and Letters of Ribeirão Preto, University of São Paulo, Ribeirão Preto 14040-901, SP, Brazil

**Keywords:** anxiety, depression, heme oxygenase 1, molecular hydrogen, neuropathy, oxidative stress, paclitaxel

## Abstract

Chemotherapy-provoked peripheral neuropathy and its associated affective disorders are important adverse effects in cancer patients, and its treatment is not completely resolved. A recent study reveals a positive interaction between molecular hydrogen (H_2_) and a heme oxygenase (HO-1) enzyme inducer, cobalt protoporphyrin IX (CoPP), in the inhibition of neuropathic pain provoked by nerve injury. Nevertheless, the efficacy of CoPP co-administered with hydrogen-rich water (HRW) on the allodynia and emotional disorders related to paclitaxel (PTX) administration has not yet been assessed. Using male C57BL/6 mice injected with PTX, we examined the effects of the co-administration of low doses of CoPP and HRW on mechanical and thermal allodynia and anxiodepressive-like behaviors triggered by PTX. Moreover, the impact of this combined treatment on the oxidative stress and inflammation caused by PTX in the amygdala (AMG) and dorsal root ganglia (DRG) were studied. Our results indicated that the antiallodynic actions of the co-administration of CoPP plus HRW are more rapid and higher than those given by each of them when independently administered. This combination inhibited anxiodepressive-like behaviors, the up-regulation of the inflammasome NLRP3 and 4-hydroxynonenal, as well as the high mRNA levels of some inflammatory mediators. This combination also increased the expression of NRF2, HO-1, superoxide dismutase 1, glutathione S-transferase mu 1, and/or the glutamate-cysteine ligase modifier subunit and decreased the protein levels of BACH1 in the DRG and/or AMG. Thus, it shows a positive interaction among HO-1 and H_2_ systems in controlling PTX-induced neuropathy by modulating inflammation and activating the antioxidant system. This study recommends the co-administration of CoPP plus HRW as an effective treatment for PTX-provoked neuropathy and its linked emotive deficits.

## 1. Introduction

The survival rates for most cancers have improved over time. This positive progress can be in part attributed to new and better treatments [1]. However, the same tendency is not detected for several of the resultant side effects of oncotherapies, which continue to have a negative effect on patients.

Paclitaxel (PTX), a taxane, is used as a first-line chemotherapeutic agent for treating several classes of cancer (ovarian, breast, lung, and pancreatic cancer) [2]. One of the most significant outcomes associated with chemotherapy utilizing PTX is the development of peripheral neuropathy, which has a significant impact on the survival rate of cancer patients and their quality of life. This symptomatology can halt or alter the course of treatment, or even stop it altogether [3].

Patients who undergo PTX chemotherapy also experience other signs such as mental diseases which can have a detrimental influence on their health and interfere with an adequate cure [4,5,6]. In addition, the severity and incidence of PTX-induced peripheral neuropathy (PIPN) and its associated comorbidities are positively correlated with the duration of treatment, which limits continued treatment. Lamentably, the current therapies aimed to reduce PIPN are limited, exhibiting modest efficacy and notable adverse effects [7].

Medicinal gaseous carbon monoxide (CO) and molecular hydrogen (H_2_) have been shown to play a relevant role in controlling different brain pathologies such as ischemia-reperfusion injury, Alzheimer’s and Parkinson’s diseases, diabetes, neuroinflammation, and multiple sclerosis [8,9,10,11]. Heme oxygenase 1 (HO-1) is a rate-limiting enzyme that catalyzes the oxidative degradation of heme into biliverdin, iron, and CO [12]. Several studies revealed that the exogenous induction of HO-1 throughout the administration of cobalt protoporphyrin IX (CoPP) has efficacious pain-killer actions under inflammatory and neuropathic pain conditions [13,14]. Furthermore, other investigations indicated that CoPP also exhibited anxiolytic and antidepressant properties [10,15,16]. Similar outcomes have been obtained with the administration of hydrogen-rich water (HRW) which also attenuated chronic pain [17,18,19] and had a critical role in modulating anxiety and depression [20,21].

Regarding the effects of CoPP and HRW administered alone in chemotherapy-induced neuropathic pain, is important to mention that almost five days of treatment with 5 mg/kg of CoPP, given one time per day, were required to reverse the allodynia (mechanical and thermal) provoked by vincristine or PTX [13,22]. Similar days of treatment with HRW at 0.30 mM, given one time per day, were requested to completely block the thermal allodynia instigated by PTX [23]. The same administration pattern and doses of CoPP or HRW administered alone were necessary to inhibit anxiodepressive-like behaviors associated with PIPN [22,23]. However, given that an inadequate treatment of neuropathic pain caused by chemotherapy might be attributed to the use of a single unique medication [7], we propose a combined treatment to effectively address PIPN as swiftly as possible. In this lane, prior works showed a positive relationship between the HO-1 and H_2_ systems in inhibiting neuropathic pain provoked by sciatic nerve injury [24,25,26]. Nonetheless, the influence of CoPP co-administered with HRW on the allodynia and emotional disorders related to PIPN has not yet been analyzed.

It has been reported that PTX induces the activation of Nod-like receptor protein 3 (NLRP3) inflammasome, and this stimulation is associated with the onset of neuropathy [27]. Therefore, PTX causes allodynia and increases the expression of NLRP3 inflammasome in the dorsal root ganglia (DRG) [27]. NLRP3 is also involved in the development of anxiodepressive-like behaviors related to diabetes and persistent inflammation [28,29]. However, the effects of CoPP combined with HRW on the expression of NLRP3 inflammasome in the DRG, an area particularly implicated in pain processing and highly affected by PTX [30], and in the AMG, a brain area particularly involved in controlling emotional behaviors [31], of PTX-injected mice have not yet been investigated.

It is well known that the development of chemotherapy-induced neuropathic pain is also influenced by oxidative stress [30]. Preceding investigations revealed that animals with PIPN exhibit elevated levels of 4-hydroxynonenal (4-HNE), an oxidative stress marker, in the DRG and prefrontal cortex [22], but its expression in the AMG has not been evaluated. Different works revealed that several antioxidants such as quercetin, 3-[4-(3-trifluoromethyl-phenyl)-piperazin-1-yl]-dihydrofuran-2-one, or berberine, an Nrf2 activator, inhibits PINP [32,33,34,35] by enhancing the expression of different antioxidant enzymes [35]. Some other treatments such as resolvin D1 and rosiglitazone (a selective agonist of PPARγ) also attenuated PIPN through the induction of the NRF2/HO-1 signaling pathway [36,37]. In addition, the exogenous activation of the antioxidant pathway by the administration of several HO-1 inducers inhibits neuropathy and the emotional disorders associated by enhancing the expression of several antioxidant enzymes such as HO-1 and SOD-1 in the DRG and/or AMG of nerve-injured mice [38] and PTX-injected animals [22], but the action of CoPP plus HRW on antioxidant protein levels has not analyzed.

In this study, using male C57BL/6 mice injected with PTX, we examined the effects of the co-administration of CoPP and HRW on the mechanical allodynia, thermal allodynia, and affective behaviors triggered by PTX to evaluate whether the effects of this combination were more effective and superior to those caused by CoPP and HRW given alone. Moreover, the modulator actions of the combined treatment of CoPP plus HRW on the inflammatory and oxidative responses incited by PTX in the DRG and AMG were also studied.

## 2. Materials and Methods

### 2.1. Animals

Male C57BL/6 mice (5–6 weeks old), obtained from Envigo Laboratories (Barcelona, Spain), were used throughout this study. They were maintained under controlled temperature and humidity (22 ± 1 °C, 66%), in a 12 h light/12 h dark cycle, with water and food accessible ad libitum. Animals were housed in polypropylene cages with wood shavings in an enriched environment that included a carton hut and cellulose pieces.

The experiments were conducted in compliance with the guidelines of the European Commission’s directive (2010/63/EC) and the Spanish Law (RD 53/2013) regulating animal research, and the procedure was approved by the local Committee of Animal Use and Care of the Autonomous University of Barcelona (ethical code is 4582, date of approval 31 March 2023). All efforts were made to minimize both the number of animals used and their suffering.

### 2.2. PTX-Derived Neuropathy

Mice were intraperitoneally injected with paclitaxel at 2 mg/kg (Tocris Bioscience, Bristol, UK) diluted in Cremophor EL ((Sigma-Aldrich, St. Louis, MO, USA)/ethanol/saline (SS, 0.9% NaCl) in a mixture of 1:1:18), every other day, for four successive days, in accordance a previous investigation [39]. The controls were injected with an identical volume of vehicle (VEH).

### 2.3. Behavioral Tests

For evaluating mechanical allodynia, animals were placed into individual methacrylate cylinders (20 cm high × 9 cm in diameter) on an elevated wire platform, through which von Frey filaments (North Coast Medical, Inc., San Jose, CA, USA) were applied in the hind paws according to the up–down paradigm [40]. Testing began with a filament of 0.4 g, and the stiffness of the subsequent filament was decided according to the reply. An Excel program (Microsoft Iberia SRL, Barcelona, Spain), which included curve fitting of the data, was used to calculate the threshold of the response. Shaking, withdrawing, or licking the hind paws were considered positive responses.

To evaluate cold allodynia, a cold plate analgesiometer (Ugo Basile, Gemonio, Italy) was employed at 4 ± 0.5 °C, and the number of hind paw elevations was recorded for 5 min.

Anxious-like behavior was evaluated by using an elevated plus maze (EPM) apparatus composed of 4 arms (5 × 35 cm) elevated to a height of 45 cm, two enclosed arms with 15 cm high walls, and two open arms. Mice were placed in the central square zone facing an open arm and were able to freely explore the maze for 5 min. The sessions were filmed by a digital camera and the number of entrances into the open and closed arms and the time spent in the open arms were quantified [41].

A tail suspension test (TST) and forced swimming test (FST) were utilized to evaluate depressant-like behaviors by which the duration of immobility of the animals was quantified according to previous works [42,43]. In the TST, the animals were suspended at 35 cm from the floor by applying adhesive tape to the tip of the tail and attaching it to an elevated surface. Their movements were filmed with a digital camera, and the immobility time was evaluated for 6 min.

In the FST, mice were placed within a transparent methacrylate cylinder measuring 25 cm in height and 10 cm in diameter, which was filled with water at a temperature of 24 ± 2 °C to a depth of 10 cm. Their activity was recorded for 6 min, and the time spent immobile for the last 4 min was evaluated.

In all tests, animals were familiarized with the testing space for 1 h before starting the proof, and experiments were conducted by researchers who were blinded to the experimental conditions.

### 2.4. Western Blot Analysis

PTX and VEH-injected mice were euthanized by cervical dislocation at day 21 post-injection and the AMG and DRG were dissected and stored at −80 °C until use. Western blot analysis was performed on proteins extracted from the AMG and DRG. Tissues were sonicated in a cold lysis buffer RIPA Buffer (Sigma-Aldrich, MO, USA). After 1 h of solubilization at 4 °C, the crude homogenates were sonicated another time for 10 s and centrifuged at 700× *g* for 20 min at 4 °C. The supernatant was combined with 4 × Laemmli loading buffer and loaded up on 4% stacking/12% separating sodium dodecyl sulfate-polyacrylamide gels. After electrophoresis, proteins were transferred onto a polyvinylidene fluoride membrane for 120 min. After that, they were blocked with phosphate-buffered saline (PBS) + 5% nonfat dry milk, PBS with Tween 20 + 5% bovine serum albumin (BSA), or Tris-buffered saline and Tween 20 + 5% nonfat dry milk or BSA for 75 min. Membranes were posteriorly incubated overnight at 4 °C with the specific primary antibody anti-NLRP3 (1:200; AG-20B-0014-C100; Adipogen Life Sciences, Epalinges, Switzerland); 4-HNE (1:150; Abcam, AB4654; Cambridge, UK); NRF2 (1:100; Cell Signaling, mAb #12721; Technology, Danvers, MA, USA); HO-1 (1:150; ADI-SPA-895; Enzo Life Sciences, New York, NY, USA); glutathione sulfur transferase M1 (GSTM1; 1:150; NBP3-15037; Novus Biologic, Littleton, CO, USA); superoxide dismutase 1 (SOD-1; 1:150; NBP2-24915; Novus Biologic, Littleton, CO, USA); BACH1 (1:100; 14018-1-AP; Proteincech, Planegg-Martinsried, Germany) or anti-glyceraldehyde-3-phosphate dehydrogenase (GAPDH; 1:5000; AB516; Merck, Billerica, MA, USA) as a loading control. After washing the membranes, they were incubated for 1 h at room temperature with the secondary antibody (GE Healthcare, Little Chalfont, UK). ECL kit reagents (GE Healthcare, Little Chalfont, UK), Chemidoc MP system (Bio-Rad, Hercules, CA, USA), and the Image-J software version 1.8.0 (National Institutes of Health, Bethesda, MD, USA) were used for the development, detection, and analysis of proteins.

### 2.5. Analysis of mRNA Levels by Quantitative Real-Time PCR (qRT-PCR)

The total RNA from the DRG and AMG was extracted utilizing RNeasy Kits for RNA Purification in accordance with the manufacturer’s instructions (Qiagen Sciences, Germantown, MD, USA). Subsequently, 1 µg of RNA from the diverse treatments was reverse transcribed for 60 min at 42 °C utilizing a RevertAid First Strand cDNA Synthesis Kit (Thermo Fisher Scientific. Waltham, MA, USA). For the Real-Time PCR analysis, the reaction was conducted in 20 µL, employing a fluorescent dye Power SYBR Green PCR Master Mix (Applied Biosystems, Foster City, CA, USA) and a mixture of 10 pmol of reverse and forward primers, whose sequences are presented in Table 1. Quantification was performed with an AriaMx Real-Time PCR System (Agilent, Santa Clara, CA, USA). The PCR cycles preceded in the following manner: an initial step of 2 min at 50 °C was followed by a denaturation step of 2 min at 95 °C, followed by 40 cycles of denaturation (15 s at 95 °C), and the annealing and extension of 60 s at 60 °C. The melting-curve analysis revealed the specificity of the amplifications. The threshold cycle, which inversely correlates with the target mRNA level, was measured as the cycle number at which the reporter fluorescent emission appears above the background threshold. To ensure that identical amounts of cDNA were added to the PCR, the GAPDH housekeeping gene was amplified. Data analysis was based on the ΔΔCT method. All PCRs were conducted in a triplicate.

### 2.6. Experimental Procedures

**Figure 1:**
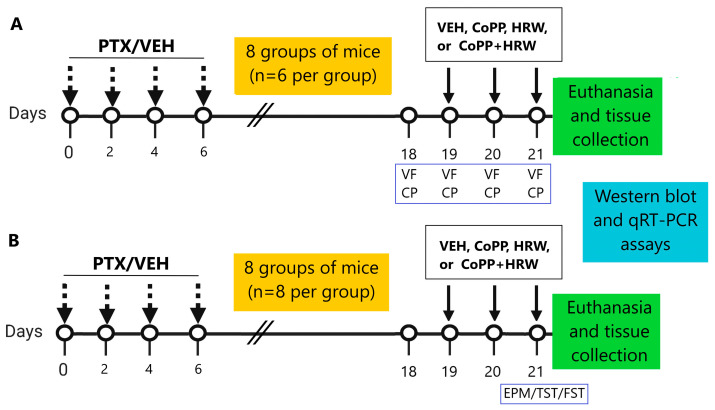
Schematic design of the experiments performed to evaluate whether the administration of CoPP (2.5 mg/kg), HRW (0.15 mM), or CoPP (2.5 mg/kg) plus HRW (0.15 mM) given intraperitoneally, twice a day, over three consecutive days, can inhibit the nociceptive responses (**A**) and/or the emotive disorders (**B**) caused by PTX. CP: cold plate test; EPM: elevated plus maze; FST: forced swimming test; PTX: paclitaxel; TST: tail suspension test; VEH; vehicle; VF: von Frey filaments.

We examined the effects of low doses of CoPP (2.5 mg/kg) or HRW (0.15 mM) given alone and combined on mechanical and thermal allodynia provoked by PTX and the anxiodepressive-like comportments associated, as described in Figure 1.

To do this, 8 groups of mice were injected twice daily with 2.5 mg/kg of CoPP or 0.15 mM of HRW or their respective VEH, alone and combined, on days 19, 20, and 21 after PTX or VEH injection. Mechanical and thermal allodynia were evaluated on the same days of treatment administration, that is, at 3 h after CoPP or CoPP plus HRW injection and at 1 h after HRW injection. HRW was injected at 2 h after CoPP administration [26] (*n* = 6 animals per group). In parallel, in 8 more groups of mice receiving the same treatments, on the same schedule, the anxious- and depressive-like comportments were evaluated at day 21 after PTX or VEH injection (*n* = 8 animals per group).

The effects of co-treatment of CoPP plus HRW on the inflammatory responses and oxidative stress caused by PTX were assessed by evaluating the expression of NLRP3, 4-HNE, NRF2, HO-1, GSTM1, and SOD-1 in the DRG and AMG by using Western blot and the levels of of IL1β, IL6, TNFα, glutamate-cysteine ligase catalytic subunit (GCLC), and the glutamate-cysteine ligase modifier subunit (GCLM) using qRT-PCR assays (*n* = 3 samples per group).

### 2.7. Drugs

CoPP acquired from Frontier Scientific (Livchem GmbH & Co., Frankfurt, Germany) was dissolved in dimethyl sulfoxide (1% in SS) and HRW was prepared using a hydrogen water generator from Hydrogen (Osmo-star Soriano S.L., Alicante, Spain). All compounds were freshly prepared before use and given intraperitoneally in a final volume of 10 mL/kg. For each treated group the respective control group received the same volume of the corresponding VEH.

### 2.8. Statistical Analyses

The statistical analysis was accomplished using the SPSS (version 28, IBM, Madrid, Spain) and Prism 8.0 (GraphPad, La Jolla, CA, USA) programs. Data are displayed as the mean values ± standard error of the mean (SEM).

A three-way repeated measures ANOVA with injection, treatment, and time as the variation factors, followed by a one-way ANOVA, and the Tuckey test were used to assess the impact of treatment with CoPP, HRW, and CoPP plus HRW on the allodynic responses caused by PTX. The actions of CoPP and HRW, alone and combined, on the emotive comportments were analyzed using a one-way ANOVA and Tuckey test. Differences in protein and mRNA levels were also calculated using a one-way ANOVA and Tuckey test. A value of *p* < 0.05 was considered significant.

## 3. Results

### 3.1. The Co-Treatment of CoPP with HRW Enhanced the Antiallodynic Effects Produced by Each Treatment Separately

**Figure 2:**
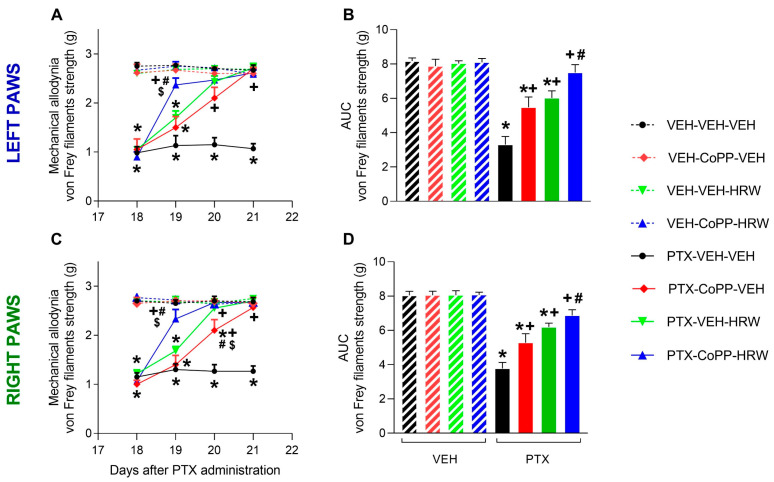
The inhibition of mechanical allodynia produced by the intraperitoneal administration of CoPP (2.5 mg/kg) and HRW (0.15 mM), alone and combined, 2 times per day, from days 19 to 21 after PTX injection, are represented. Data are presented as the von Frey filaments strength (g) on the left (**A**) and right (**C**) paws and their respective AUC to examine the global effect of these treatments (**B**,**D**). In all figures, symbols show significant differences vs. subjects given * VEH-VEH-VEH, VEH-CoPP-VEH, VEH-VEH-HRW or VEH-CoPP-HRW, + vs. PTX-VEH-VEH, # vs. PTX-CoPP-VEH and $ vs. PTX-HRW-VEH (*p* < 0.05, one-way ANOVA and Tukey test). Mean values ± SEM of 6 animals per group.

**Figure 3:**
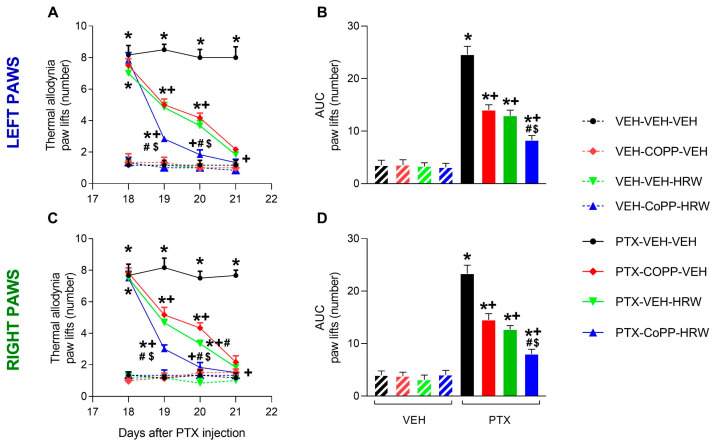
The inhibition of thermal allodynia produced by the intraperitoneal administration of CoPP (2.5 mg/kg) and HRW (0.15 mM), alone and combined, 2 times per day, from days 19 to 21 after PTX injection, are represented. Data are presented as the paw lifts (number) in the left (**A**) and right (**C**) paws and their respective AUC to examine the global effect of these treatments (**B**,**D**). In all figures, symbols show significant differences vs. subjects given * VEH-VEH-VEH, VEH-CoPP-VEH, VEH-VEH-HRW or VEH-CoPP-HRW, + vs. PTX-VEH-VEH, # vs. PTX-CoPP-VEH and $ vs. PTX-HRW-VEH (*p* < 0.05, one-way ANOVA and Tukey test). Mean values ± SEM of 6 animals per group.

We evaluated the effects of CoPP (2.5 mg/kg) and HRW (0.15 mM) given intraperitoneally, alone and combined, on mechanical (Figure 2) and thermal allodynia (Figure 3) caused by PTX from day 19 to 21 after injection in both hind paws. In both tests, significant effects of injection (*p* < 0.001), treatment *p* < 0.001), time (*p* < 0.001), and interactions between injection and treatment (*p* < 0.001), injection and time (*p* < 0.001), treatment and time (*p* < 0.001), and between them were revealed by the three-way ANOVA repeated measures in the left and right paws.

Our finding showed that the low threshold of paw withdrawal from von Frey filaments provoked by PTX on day 18 from injection (*p* < 0.001, one-way ANOVA vs. VEH-VEH-VEH) was completely reversed after two or three days of treatment with CoPP and HRW administered alone in both hind paws (Figure 2A,C). Remarkably, a complete inhibition of mechanical allodynia triggered by PTX was observed on the first day of the co-treatment of CoPP with HRW in both hind paws (*p* < 0.001; one-way ANOVA). Additionally, the antiallodynic effects of the concurrent administration of CoPP and HRW on the first day of treatment were greater than those produced by both compounds given alone (*p* < 0.001; one-way ANOVA). The analysis of the area under curve (AUC) confirmed the partial and complete inhibition of mechanical allodynia in the left (Figure 2B) and right paws (Figure 2D) of mice given CoPP or HRW administered alone and combined, respectively (*p* < 0.001; one-way ANOVA; vs. their respective VEH treated animals).

In the cold plate test, CoPP and HRW administered alone also progressively reduced thermal allodynia incited by PTX throughout the three days of treatment in the left (Figure 3A) and right paws (Figure 3C; *p* < 0.001; one-way ANOVA vs. their respective VEH-treated animals), reaching a full reversal on the third day of treatment. Remarkably, the complete reversal of thermal allodynia was accomplished after two days of treatment of CoPP plus HRW and its effects were also greater than those performed by each of them after one or two days of treatment (*p* < 0.001, one-way ANOVA). The analysis of the AUC corroborated that the inhibition of PTX-caused thermal allodynia was higher in the left (Figure 3B) and right paws (Figure 3D) of mice treated with CoPP plus HRW than those administered with each of them separately (*p* < 0.001; one-way ANOVA).

In both tests and paws analyzed, CoPP or HRW given alone or merged did not cause any changes in animals given the VEH.

### 3.2. The Concurrent Administration of CoPP and HRW Normalized the Anxious- and Depressant-like Comportments Linked to PIPN

**Figure 4:**
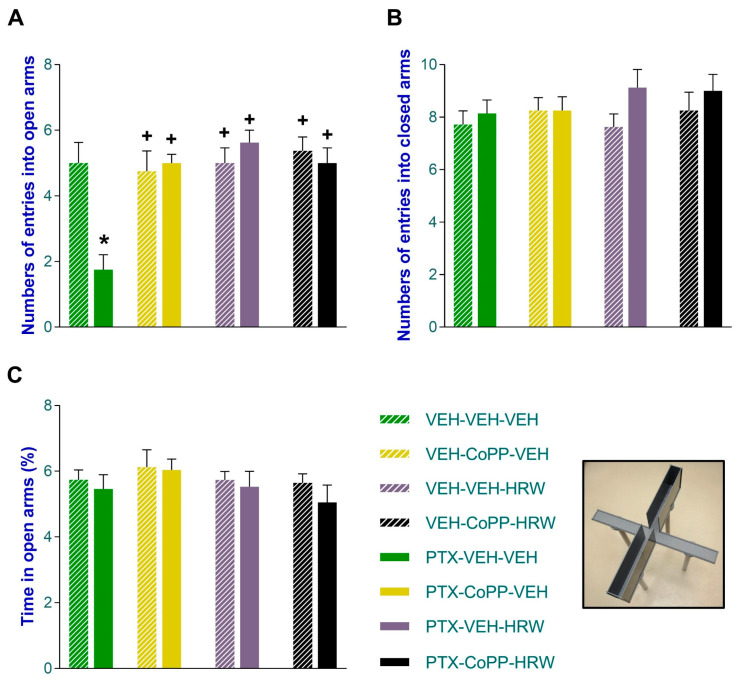
The inhibition of anxious-like behaviors associated with PIPN induced by the intraperitoneal administration of CoPP (2.5 mg/kg) or HRW (0.15 mM), alone and combined, given 2 times per day over three consecutive days, are represented. The effects of CoPP, HRW, CoPP plus HRW, or VEH in animals given the VEH are also shown. The number of entrances to the open arms (**A**) and closed arms (**B**) and the proportion of time passed in the open arms (**C**) of the EPM are represented. In all graphs, * signifies significant differences vs. animals treated with VEH-VEH-VEH and + vs. animals treated with PTX-VEH-VEH (*p* < 0.05, one-way ANOVA and Tuckey test). Data are expressed as mean values ± SEM of 8 animals per group.

In accordance with the literature, PIPN was accompanied by anxious- and depressive-like comportments displayed on day 21 after injection. The anxious-like behavior manifested in the low number of entrances into the open arms of the EPM (*p* < 0.021, one-way ANOVA; vs. VEH-VEH-VEH treated mice; Figure 4A) was completely reversed by the administration of CoPP and HRW, given alone and combined, for three consecutive days. The injection of PTX did not change the number of entries into the closed arms (Figure 4B) nor the proportion of time spent in the open arms (Figure 4C) and neither treatment altered these behaviors.

**Figure 5:**
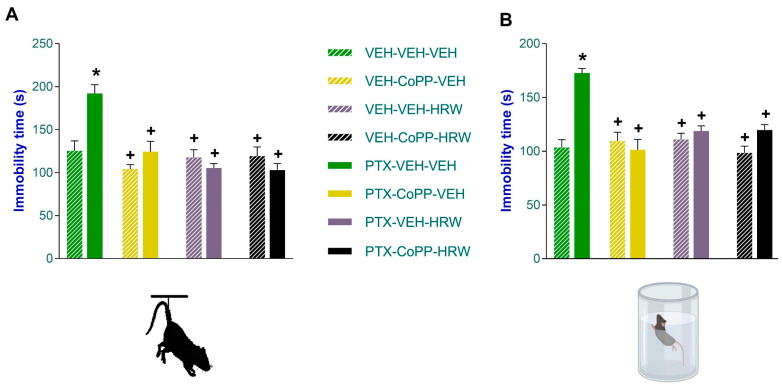
The inhibition of depressant-like behaviors associated with PIPN induced by the intraperitoneal administration of CoPP (2.5 mg/kg) or HRW (0.15 mM), alone and combined, given 2 times per day over three consecutive days, are represented. The effects of CoPP, HRW, CoPP plus HRW, or VEH in animals given the VEH are also shown. In the TST (**A**) and FST (**B**), the time that the animals remain immobile (s) is represented. In both graphs, * signifies significant differences vs. animals treated with VEH-VEH-VEH and + vs. animals treated with PTX-VEH-VEH (*p* < 0.05, one-way ANOVA and Tuckey test). Data are expressed as mean values ± SEM of 8 animals per group.

The impact of the co-treatment of CoPP plus HRW on the depressive-like behaviors caused by chemotherapy was evaluated in the TST (Figure 5A) and FST (Figure 5B) on day 21 after PTX injection. Both tests showed that the increased immobility time observed in PTX-injected animals treated with VEH was completely normalized by the administration of CoPP or HRW, given separately or in combination, for three consecutive days (*p* < 0.001, one-way ANOVA vs. VEH-VEH-VEH treated mice).

The findings show that all of these treatments had anxiolytic and antidepressant effects on PTX-injected animals.

### 3.3. The Effects of Combining CoPP with HRW on the Levels of NLRP3, 4-HNE, NRF2, HO-1, GSTM1, SOD-1, and BACH1 in the DRG and AMG of PTX-Injected Mice

**Figure 6:**
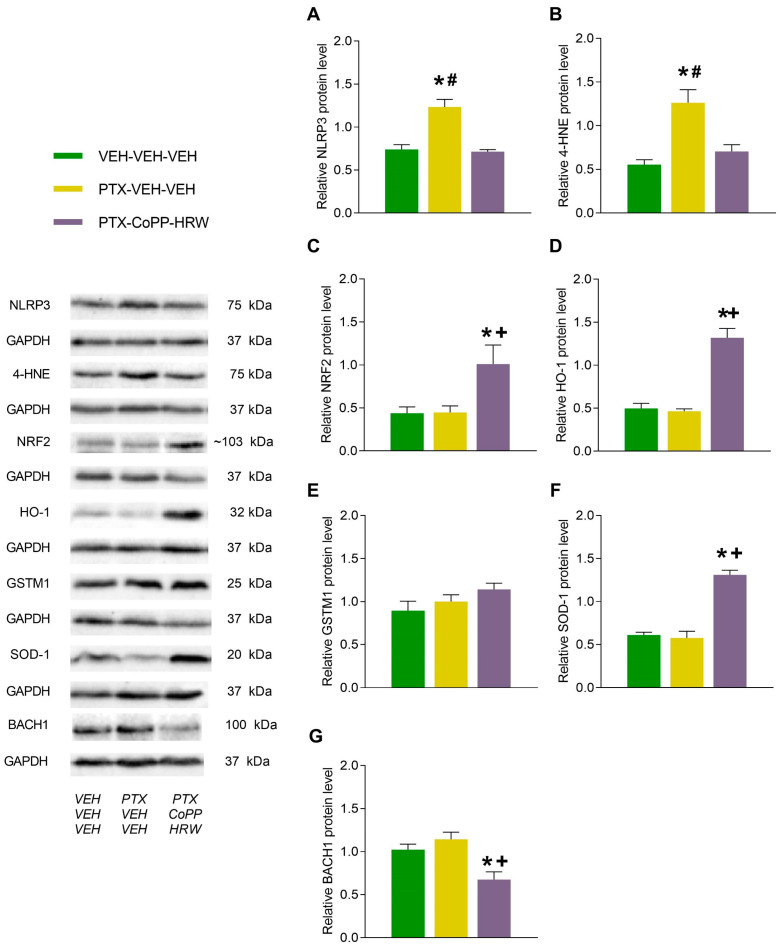
The effects of CoPP combined with HRW on the protein levels of NLRP3, 4-HNE, NRF2, HO-1, GSTM1, SOD-1, and BACH1 in the DRG of PTX-injected mice. This combined treatment reversed the up-regulation of NLRP3 (**A**) and 4-HNE (**B**), increased the protein levels of NRF2 (**C**), HO-1 (**D**), and SOD-1 (**F**), and decreased those of BACH1 (**G**) in the DRG of PTX-injected mice. No changes in GSTM1 levels (**E**) were observed. VEH-injected mice treated with VEH plus VEH were used as controls. In all graphs, symbols denote significant changes vs., * VEH-VEH-VEH treated mice, + vs. PTX-injected animals treated with VEH-VEH and # vs. PTX-injected mice treated whit CoPP-HRW (*p* < 0.05; one-way ANOVA and Tukey test). Data are presented as mean values ± SEM of 3 samples/groups.

Our results revealed increased levels of NLRP3 (*p* < 0.001 vs. VEH-VEH-VEH treated animals; Figure 6A) and 4-HNE (*p* < 0.006 vs. VEH-VEH-VEH treated animals, Figure 6B) provoked by PTX in the DRG, which were completely stabilized by the co-treatment of CoPP plus HRW. This mixed treatment also activated the antioxidant system by increasing the expression of NRF2 (*p* < 0.030 vs. VEH-VEH-VEH and PTX-VEH-VEH treated animals; Figure 6C), HO-1 (*p* < 0.001 vs. VEH-VEH-VEH and PTX-VEH-VEH treated animals; Figure 6D), and SOD-1 (*p* < 0.002 vs VEH-VEH-VEH and PTX-VEH-VEH treated animals; Figure 6F) while decreasing BACH1 protein levels (*p* < 0.015 vs VEH-VEH-VEH and PTX-VEH-VEH treated animals; Figure 6G) in the DRG. The protein levels of GSTM1 remained intact (Figure 6E).

**Figure 7:**
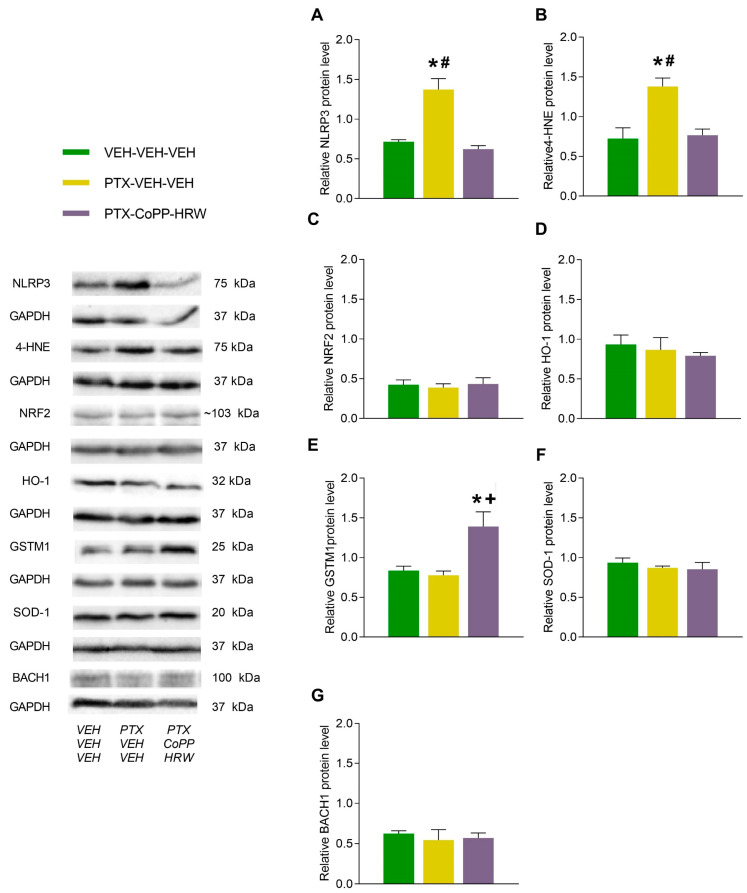
The effects of CoPP combined with HRW on the protein levels of NLRP3, 4-HNE, NRF2, HO-1, GSTM1, SOD-1, and BACH1 in the AMG of PTX-injected mice. This combined treatment reversed the up-regulation of NLRP3 (**A**) and 4-HNE (**B**) and increased the protein levels of GSTM1 (**E**) in the AMG of PTX-injected mice. No changes in NRF2 (**C**), HO-1 (**D**), SOD-1 (**F**), and BACH1 (**G**) were identified. VEH-injected mice treated with VEH plus VEH were used as controls. In all graphs, symbols denote significant changes vs., * VEH-VEH-VEH treated mice, + vs. PTX-injected animals treated with VEH-VEH and # vs. PTX-injected mice treated with CoPP-HRW (*p* < 0.05; one-way ANOVA, followed by the Tukey test). Data are presented as mean values ± SEM of 3 samples/group.

PTX injection also enhanced NLRP3 (*p* < 0.001, one-way ANOVA vs. VEH-VEH-VEH treated subjects; Figure 7A) and 4-HNE levels (*p* < 0.009, one-way ANOVA vs. VEH-VEH-VEH treated subjects; Figure 7B) in the AMG. The co-treatment of CoPP with HRW normalized the NLRP3 inflammasome activation and oxidative stress caused by PTX in the AMG and further augmented GSTM1 expression in this brain area (*p* < 0.017; one-way ANOVA vs VEH-VEH-VEH and PTX-VEH-VEH treated animals; Figure 7E). Neither the PTX injection nor CoPP plus HRW administration changed the protein levels of NRF2 (Figure 7C), HO-1 (Figure 7D), SOD-1 (Figure 7F), or BACH1 (Figure 7G) in the AMG.

### 3.4. The Effects of CoPP Combined with HRW on the mRNA Levels of IL-1β, IL-6, TNFα, CGLC, and GCLM in the DRG and AMG of PTX-Injected Mice

**Figure 8:**
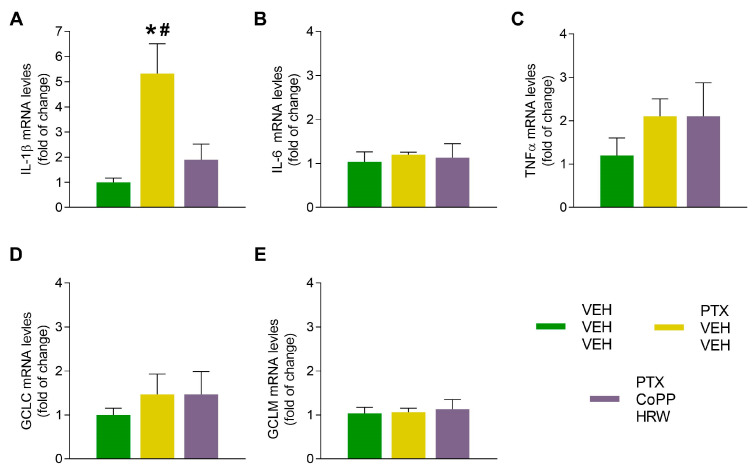
The effects of CoPP combined with HRW on the mRNA levels of IL-1β, IL6, TNFα, GCLC, and GCLM in the DRG of PTX-injected mice. This combined treatment reversed the up-regulation of IL-1B (**A**). No changes in the IL-6 (**B**), TNFα (**C**), GCLC (**D**), or GCLM (**E**) were identified. VEH-injected mice treated with VEH plus VEH were used as controls. In all graphs, symbols denote significant changes vs., * VEH-VEH-VEH treated mice (*p* < 0.05; one-way ANOVA and # vs. PTX-injected mice treated with CoPP-HRW, followed by the Tukey test). Data are presented as mean values ± SEM of 3 samples/group.

**Figure 9:**
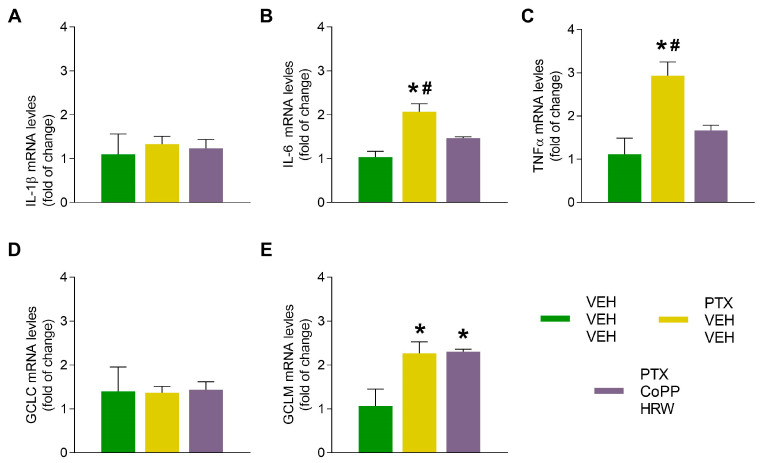
The effects of CoPP combined with HRW on the mRNA levels of IL-1β, IL6, TNFα, GCLC, and GCLM in the AMG of PTX-injected mice. This combined treatment reversed the up-regulation of IL-6 (**B**) and TNFα (**C**) and maintained the increased expression of GCLM (**E**) provoked by PTX. No changes in IL-1β (**A**) and GCLC (**D**) were identified. VEH-injected mice treated with VEH plus VEH were used as controls. In all graphs, symbols denote significant changes vs., * VEH-VEH-VEH treated mice and # vs. PTX-injected mice treated with CoPP-HRW, followed by the Tukey test. Data are presented as mean values ± SEM of 3 samples/groups.

Our results revealed that PTX increased the mRNA levels of the proinflammatory cytokines IL-1β (*p* < 0.017 vs. VEH-VEH-VEH treated animals; Figure 8A) in the DRG and those of IL-6 (*p* < 0.004 vs. VEH-VEH-VEH treated animals; Figure 9B) and TNFα (*p* < 0.011 vs. VEH-VEH-VEH treated animals; Figure 9C) in the AMG. The administration of CoPP combined with HRW inhibited the high mRNA levels of these genes. These data reinforce the increased protein levels of NLRP3 incited by PTX as well as its inhibition with the administration of CoPP plus HRW in both tissues.

Regarding antioxidant proteins, PTX injection increases the mRNA levels of GCLM in the AMG as a defensive mechanism to combat oxidative stress, and these high levels were maintained in CoPP plus HRW-treated animals (*p* < 0.029 vs. VEH-VEH-VEH treated animals; Figure 9E).

## 4. Discussion

This study demonstrates that the concurrent administration of CoPP and HRW effectively inhibits PIPN in mice. This combined treatment potentiates the antiallodynic actions of each treatment and preserves their antidepressant and anxiolytic properties. These effects are primarily attributed to the inhibition of NLRP3 inflammasome activation and oxidative stress, as well as by the induction of the antioxidant system in the DRG and/or AMG. Our findings reveal the rapidity and effectiveness of this mixed therapy and suggest its use for treating the allodynia and mood disorders that accompany PIPN.

Chemotherapy-induced peripheral neuropathy is one of the most common and harmful side effects of the anticancer drug PTX which causes significant discomfort to cancer patients. Currently, there are no effective treatments for PIPN. Indeed, whereas several studies prove that opioids and NMDA receptor antagonists are not effective against PIPN [44], others reveal that selective T-type calcium channel blockers, CXCR1/CXCR2 inhibitors, cannabidiol, melatonin, and vitamin E can alleviate PIPN [45,46,47,48,49]. However, as a consequence of their side effects, most of these drugs have restricted use for the treatment of PIPN. Likewise, given the delicate emotional state of patients receiving PTX as a chemotherapeutic agent, the fact that the possible anxiolytic or antidepressant properties of these drugs have not been thoroughly evaluated may hinder their applicability. In this study, we propose the use of a combination approach to achieve a more global and efficient treatment for PIPN and its associated comorbidities.

Our data reveal that the combined administration of CoPP and HRW inhibits allodynia more rapidly than either treatment administered separately. Indeed, only one or two days of combined therapy were needed to completely reduce mechanical and thermal allodynia triggered by PTX, while almost three days of simple therapy with CoPP or HRW were required. In addition, the antiallodynic actions performed by the combination of CoPP plus HRW were higher than those produced by each of them given separately. These results reveal a synergy between HO-1 and H_2_ systems in inhibiting PIPN, which agrees with the demonstrated positive interplay among them for the inhibition of the allodynia and hyperalgesia caused by nerve injury in rodents [26], and with the participation of the HO-1/CO pathway in the analgesic effects of HRW in animals with nerve-injury-induced neuropathic pain [24,25]. Our study also indicated that the effect of the administration of low doses of CoPP or HRW, given alone at two times per day, is more effective than that of the administration of high doses of these compounds, given one time per day. Indeed, three days of treatment with CoPP, administered at 2.5 mg/kg two times per day, is sufficient to reverse mechanical allodynia whereas five days of treatment with a double dose of CoPP (5 mg/kg), administered one time per day, were required. Our data reveal that the co-administration of CoPP with HRW, two times per day, maximizes the effects of both treatments and might be a suitable alternative for the prompt and effective management of allodynia related to PTX-induced chemotherapy.

It is well recognized that pain is accompanied by severe emotional disorders that can last a long time after the end of PTX treatment and notability affects the quality of life of cancer patients [50]. It has been reported that the administration of reactive oxygen species (ROS) scavengers or the activation of the antioxidant system mitigates the development of neuropathic pain and its associated affective disturbances in different animal pain models [36,38,51]. In the same way, treatment with HRW also reduced the nociceptive and anxiodepressive-like behaviors related to inflammatory and neuropathic pain provoked by sciatic nerve injury [26,52]. In addition, recent studies reveal the anxiolytic and antidepressant properties of CoPP [10,15,16,53] as well as those produced by HRW alone [20,21] in the absence of chronic pain. However, the effects of their combined administration in PTX-injected mice have not been evaluated.

Our findings reveal that three days of treatment with low doses of CoPP (2.5 mg/kg) or HRW (0.15 mM), given alone or concurrently twice daily, all inhibit the anxiodepressive-like behaviors accompanying PIPN. These data differ from the five days of treatment with CoPP (5 mg/kg), administered alone one time per day, required for inhibiting the anxiety- and depressive-like behaviors associated with PIPN [22]. This shows the major effectiveness of CoPP, when administered two times per day instead of one, in the modulation of the emotional deficits caused by PTX. Our data also demonstrate that the combined treatment of CoPP plus HRW maintains the reversion of emotional disorders performed by both compounds given alone. Indeed, CoPP and HRW alone and combined analogously reversed the low number of visits to the open arms observed in the EPM test and the high immobility time recorded in the TST and FST of PTX-injected mice treated with VEH. Moreover, the number of entries into the closed arms of the EPM test, a variable associated with locomotor activity, was not altered by any treatment, proving that the observed anxiolytic actions were not influenced by locomotor activity. These effects are in line with those produced by the adeno-associated virus-mediated overexpression of HO-1 in mice [16]. In summary, our findings demonstrate, for the first time, the efficacy and rapid actions mediated by the administration of low doses of CoPP and HRW, alone and combined, in alleviating emotional symptoms initiated by PTX. These results have high importance since most conventional therapies such as some anticonvulsants or opioids, have moderate or low efficacy in eradicating neuropathy and/or linked anxiodepressive-like behaviors [54].

The primary mechanisms underlying the effects induced by the combined therapy of CoPP and HRW were evaluated by examining its impact on the oxidative stress and inflammasome NLRP3 activation induced by PTX in the DRG and AMG. Numerous studies have demonstrated that the production of ROS is a significant contributor to the progression of neuropathic pain induced by chemotherapy [55,56]. Alternatively, the NRF2 transcription factor serves as a pivotal regulator of the endogenous antioxidant defense [57] and the activation of its downstream target genes, such as HO-1, SOD-1, and GSTM1, is crucial for regulating neuropathy induced by chemotherapy [58]. HO-1 is also considered one of the main antioxidant defense mechanisms playing pivotal roles in the CNS, such as neuroprotection which could also be involved in preventing neuropathic pain. However, high HO-1 expression has also been associated with neurodegeneration and neuronal damage processes, thus revealing the dual role played by this enzyme in the nervous system [59,60]. Our results confirmed an up-regulation of 4-HNE in the DRG and AMG of PTX-injected mice, which supports the oxidative stress caused by this chemotherapeutic agent [23]. Interestingly, the combination of CoPP and HRW stabilized the increased expression of 4-HNE, showing the powerful anti-oxidative effects of this combined therapy in PTX-injected mice. Furthermore, treatment with CoPP and HRW was able to up-regulate the protein levels of NRF2 and of the antioxidant enzymes HO-1 and SOD-1 in the DRG, and those of GSTM1 as well the mRNA levels of GCLM in the AMG. Supporting these findings, similar results were obtained with the administration of CoPP or HRW given alone, which also reduced the up-regulation of 4-HNE provoked by PTX and increased HO-1 and SOD-1 levels in the DRG, and those of GSTM1 in the prefrontal cortex [22,23]. In addition, the comparable increase in SOD-1 and HO-1 levels observed in the DRG of PTX-injected animals treated with CoPP plus HRW revealed the similar effect produced by this combined treatment on the activation of both antioxidant enzymes. Furthermore, considering that CoPP is a great repressor of BACH1 by driving its degradation through the ubiquitin–proteasome system, this allows Maf-Maf, NRF2-Maf, and other activating heterodimers to occupy the heme-responsive element sites in the HO-1 promoter, leading to increased HO-1 expression [61]. To know if CoPP plus HRW can increase HO-1 levels through the BACH1 pathway, we evaluated the expression of BACH1 in the DRG and AMG. Our results revealed that in addition to the increased expression of Nrf2, the decreased levels of BACH1 caused by CoPP plus HRW in the DRG may also be responsible for the upregulation of HO-1 produced by CoPP plus HRW in the DRG of PTX-injected mice.

Our results confirmed the antioxidant capabilities of CoPP combined with HRW and further showed the different impacts of this combined treatment on the expression of antioxidant enzymes in the central and peripheral nervous system. Consistent with these findings, similar results have been demonstrated for the aging cerebral cortex and hippocampus, where HO-1 regulates brain oxysterols only in the cortex controlled by the early transcriptional factor Egr-1 [62]. Furthermore, considering the increased protein levels of NRF2, HO-1, and SOD-1 in the DRG and GSTM1 in the AMG together with the high mRNA levels of GCLM in this brain area, we speculate that, while NRF2, HO-1, and SOD-1 appear to be more involved in the control of sensorial reactions, GSTM1 and GCLM appear to be more implicated in the regulation of emotional responses altered by PTX. Therefore, the antiallodynic and anxiolytic/antidepressant effects produced by CoPP plus HRW might be caused by activating the endogenous antioxidant system in the DRG and AMG. These findings are substantiated by the capability of several HO-1 inducers and HRW to inhibit chronic pain and mental disorders subsequent to peripheral inflammation or sciatic nerve injury [26,38,52,63], as well as by their potent antioxidant properties and the implication of oxidative stress in regulating psychiatric disorders [64,65]. In accordance with these findings, the administration of other natural or synthetic antioxidant molecules, such as carvacrol, also exerts neuroprotective effects in Parkinson’s disease via anti-inflammatory and antioxidant mechanisms by triggering the Nrf2/HO-1 pathway [66]. Derivatives of 3,3-Disubstituted-3H-benzofuran-2-one also exhibit antioxidant properties due to their capacity to reduce intracellular ROS levels and prevent cell death by enhancing HO-1 expression in a cellular model of neurodegeneration [67]. In addition, the administration of daidzein also reduces PIPN by down-regulating TRPV1/P2Y and up-regulating the Nrf2/HO-1 signaling pathways [68].

Many works revealed the implication of the NLRP3 inflammasome in the pathology of several peripheral disorders including arthritis, neuropathy, and inflammatory pain [69,70,71]. In the context of chemotherapy, it is also established that the activation of the NLRP3 inflammasome triggered by oxidative stress is a crucial contributor to the development of PIPN [27]. This process results in the release of pro-inflammatory factors such as IL-1β and IL-18, which increase the excitability of sensory neurons in the DRG, which then transmit these signals to the spinal cord and the brain [72]. Consequently, elevated levels of NLRP3 and some cytokines were observed in the DRG, sciatic nerves, and/or spinal cords of animals suffering from PTX-, oxaliplatin-, or vincristine-induced peripheral neuropathy [69,73] as well as in the AMG of animals with neuropathic pain caused by chronic constriction of the sciatic nerve [38]. In accordance, mice injected with PTX also showed increased protein levels of NLRP3 in the DRG and AMG, confirming the involvement of this inflammasome in this type of chemotherapy. These findings were further reinforced by the increased mRNA levels of IL-1β and those of IL-6 and TNFα observed in the DRG and AGM of PTX-injected mice. Moreover, the co-treatment of CoPP plus HRW decreased these up-regulations in both areas, revealing the central and peripheral anti-inflammatory actions of this combined treatment in PTX-injected mice. Considering that blocking the NLRP3 activation pathway alleviates chemotherapy-induced neuropathy [74] and that the dysregulation of NLRP3 inflammasome in some brain regions participates in the development of mood disorders [75], we hypothesized that the normalization of NLRP3 up-regulation and the inhibition of IL-1β, IL-6, and TNFα overexpression in the DRG and/or AMG might also take part in the inhibition of the allodynia and anxiodepressive-like behaviors which is caused by CoPP plus HRW during PTX-induced chemotherapy. In agreement, several HO-1 inducers palliate inflammatory pain and the emotional disorders associated through regulating NLRP3 inflammasome [63]. Therefore, the dual regulation of the inflammatory and antioxidant paths made by the combined therapy of CoPP and HRW might explain the great efficacy of its co-treatment in inhibiting PIPN and its linked emotional illnesses.

## 5. Conclusions

To sum up, this study provides evidence that the combination of CoPP and HRW could be a fast and efficient therapy for PIPN. Our results demonstrate that CoPP combined with HRW inhibited the neuropathy, anxious-, and depressant-like behaviors generated by PTX. These effects might be a consequence of the potentiation of the antioxidant system, the inhibition of NLRP3 inflammasome activation, and the synthesis of pro-inflammatory mediators produced by this combined treatment in the DRG and/or AMG of PTX-injected mice. This study supports the importance of HO-1 and H_2_ systems in modulating PIPN and its accompanying mood disorders, and further suggests CoPP combined with HRW as a safe and effective alternative for its treatment.

## Figures and Tables

**Table 1:** Mouse primers used for qRT-PCR.

Gene Product	Forward Primer (5′-3′)	Reverse Primer (5′-3′)
*Il1b*	CTGGTGTGTGACGTTCCCATTA	CCGACAGCACGAGGCTTT
*Il6*	CCTACCCCAATTTCCAATGCT	TATTTTCTGACCACAGTGAGGAATG
*Tnfa*	CATCTTCTCAAAATTCGAGTGACAA	TGGGAGTAGACAAGGTACAACCC
*Gclc*	TTACCGAGGCTACGTGTCAGAC	TATCGATGGTCAGGTCGATGTC
*Gclm*	AATCAGCCCCGATTTAGTCAGG	CCAGCGTGCAACTCCAAGGAC
*Gapdh*	CGACTTCAACAGCAACTCCCACTCTTCC	TGGGTGGTCCAGGGTTTCTTACTCCTT

## Data Availability

The data presented in this study are available in the article.

## References

[1] Flatters S.J.L., Dougherty P.M., Colvin L.A. (2017). Clinical and preclinical perspectives on Chemotherapy-Induced Peripheral Neuropathy (CIPN): A narrative review. Br. J. Anaesth..

[2] Stage T.B., Bergmann T.K., Kroetz D.L. (2018). Clinical Pharmacokinetics of Paclitaxel Monotherapy: An Updated Literature Review. Clin. Pharmacokinet..

[3] Engvall K., Gréen H., Fredrikson M., Lagerlund M., Lewin F., Åvall-Lundqvist E. (2022). Impact of persistent peripheral neuropathy on health-related quality of life among early-stage breast cancer survivors: A population-based cross-sectional study. Breast Cancer Res. Treat..

[4] Seretny M., Currie G.L., Sena E.S., Ramnarine S., Grant R., MacLeod M.R., Colvin L.A., Fallon M. (2014). Incidence, prevalence, and predictors of chemotherapy-induced peripheral neuropathy: A systematic review and meta-analysis. Pain.

[5] Banach M., Juranek J.K., Zygulska A.L. (2016). Chemotherapy-induced neuropathies—A growing problem for patients and health care providers. Brain Behav..

[6] Staff N.P., Grisold A., Grisold W., Windebank A.J. (2017). Chemotherapy-induced peripheral neuropathy: A current review. Ann. Neurol..

[7] Gewandter J.S., Kleckner A.S., Marshall J.H., Brown J.S., Curtis L.H., Bautista J., Dworkin R.H., Kleckner I.R., Kolb N., Mohile S.G. (2020). Chemotherapy-induced peripheral neuropathy (CIPN) and its treatment: An NIH Collaboratory study of claims data. Support. Care Cancer.

[8] Jiao Y., Yu Y., Li B., Gu X., Xie K., Wang G., Yu Y. (2020). Protective effects of hydrogen-rich saline against experimental diabetic peripheral neuropathy via activation of the mitochondrial ATP-sensitive potassium channel channels in rats. Mol. Med. Rep..

[9] Lee D., Choi J.I. (2021). Hydrogen-Rich Water Improves Cognitive Ability and Induces Antioxidative, Antiapoptotic, and Anti-Inflammatory Effects in an Acute Ischemia-Reperfusion Injury Mouse Model. BioMed Res. Int..

[10] Baskin V., Eroglu E., Harmanci N., Erol K. (2022). Antinociceptive, anxiolytic, and depression-like effects of hydrogen sulfide, nitric oxide, and carbon monoxide in rats and the role of opioidergic and serotonergic systems in antinociceptive activity. Fundam. Clin. Pharmacol..

[11] Lin Y.T., Shi Q.Q., Zhang L., Yue C.P., He Z.J., Li X.X., He Q.J., Liu Q., Du X.B. (2022). Hydrogen-rich water ameliorates neuropathological impairments in a mouse model of Alzheimer’s disease through reducing neuroinflammation and modulating intestinal microbiota. Neural ReGrn Res..

[12] Abraham N.G., Kappas A. (2008). Pharmacological and clinical aspects of heme oxygenase. Pharmacol. Rev..

[13] Shen Y., Zhang Z.J., Zhu M.D., Jiang B.C., Yang T., Gao Y.J. (2015). Exogenous induction of HO-1 alleviates vincristine-induced neuropathic pain by reducing spinal glial activation in mice. Neurobiol. Dis..

[14] Zhou Y.Q., Liu D.Q., Chen S.P., Chenm N., Sun J., Wang X.M., Cao F., Tian Y.K., Ye D.W. (2020). Nrf2 activation ameliorates mechanical allodynia in paclitaxel-induced neuropathic pain. Acta Pharmacol. Sin..

[15] Cazuza R.A., Pol O., Leite-Panissi C.R.A. (2018). Enhanced expression of heme oxygenase-1 in the locus coeruleus can be associated with anxiolytic-like effects. Behav. Brain Res..

[16] Luo Y., Ullah R., Wang J., Du Y., Huang S., Meng L., Gao Y., Gong M., Galaj E., Yin X. (2021). Exogenous carbon monoxide produces rapid antidepressant- and anxiolytic-like effects. Front. Pharmacol..

[17] Ge Y., Wu F., Sun X., Xiang Z., Yang L., Huang S., Lu Z., Sun Y., Yu W.F. (2014). Intrathecal infusion of hydrogen-rich normal saline attenuates neuropathic pain via inhibition of activation of spinal astrocytes and microglia in rats. PLoS ONE.

[18] Kawaguchi M., Satoh Y., Otsubo Y., Kazama T. (2014). Molecular hydrogen attenuates neuropathic pain in mice. PLoS ONE.

[19] Wang H., Huo X., Chen H., Li B., Liu J., Ma W., Wang X., Xie K., Yu Y., Shi K. (2018). Hydrogen-Rich Saline Activated Autophagy via HIF-1 α Pathways in Neuropathic Pain Model. BioMed Res. Int..

[20] Zhang Y., Su W.J., Chen Y., Wu T.Y., Gong H., Shen X.L., Wang Y.X., Sun X.J., Jiang C.L. (2016). Effects of hydrogen-rich water on depressive-like behavior in mice. Sci. Rep..

[21] Masuda K., Tanaka Y., Kanehisa M., Ninomiya T., Inoue A., Higuma H., Kawashima C., Nakanishi M., Okamoto K., Akiyoshi J. (2017). Natural reduced water suppressed anxiety and protected the heightened oxidative stress in rats. Neuropsychiatr. Dis. Treat..

[22] Roch G., Batallé G., Bai X., Pouso-Vázquez E., Rodríguez L., Pol O. (2022). The Beneficial Effects of Heme Oxygenase 1 and Hydrogen Sulfide Activation in the Management of Neuropathic Pain, Anxiety- and Depressive-like Effects of Paclitaxel in Mice. Antioxidants.

[23] Martínez-Martel I., Bai X., Batallé G., Pol O. (2022). New Treatment for the Cognitive and Emotional Deficits Linked with Paclitaxel-Induced Peripheral Neuropathy in Mice. Antioxidants.

[24] Chen Q., Chen P., Zhou S., Yan X., Zhang J., Sun X., Yuan H., Yu W. (2013). Hydrogen-rich saline attenuated neuropathic pain by reducing oxidative stress. Can. J. Neurol. Sci..

[25] Chen Y., Chen H., Xie K., Liu L., Li Y., Yu Y., Wang G. (2015). H2 Treatment Attenuated Pain Behavior and Cytokine Release Through the HO-1/CO Pathway in a Rat Model of Neuropathic Pain. Inflammation.

[26] Martínez-Serrat M., Martínez-Martel I., Coral-Pérez S., Bai X., Batallé G., Pol O. (2022). Hydrogen-Rich Water as a Novel Therapeutic Strategy for the Affective Disorders Linked with Chronic Neuropathic Pain in Mice. Antioxidants.

[27] Jia M., Wu C., Gao F., Xiang H., Sun N., Peng P., Li J., Yuan X., Li H., Meng X. (2017). Activation of NLRP3 inflammasome in peripheral nerve contributes to paclitaxel-induced neuropathic pain. Mol. Pain.

[28] Zhai Y., Meng X., Ye T., Xie W., Sun G., Sun X. (2018). Inhibiting the NLRP3 Inflammasome Activation with MCC950 Ameliorates Diabetic Encephalopathy in db/db Mice. Molecules.

[29] Arioz B.I., Tastan B., Tarakcioglu E., Tufekci K.U., Olcum M., Ersoy N., Bagriyanik A., Genc K., Genc S. (2019). Melatonin Attenuates LPS-Induced Acute Depressive-like Behaviors and Microglial NLRP3 Inflammasome Activation Through the SIRT1/Nrf2 Pathway. Front. Immunol..

[30] Starobova H., Vetter I. (2017). Pathophysiology of chemotherapy-induced peripheral neuropathy. Front. Mol. Neurosci..

[31] Neugebauer V., Mazzitelli M., Cragg B., Ji G., Navratilova E., Porreca F. (2020). Amygdala, neuropeptides, and chronic pain-related affective behaviors. Neuropharmacology.

[32] Sałat K., Cios A., Wyska E., Sałat R., Mogilski S., Filipek B., Więckowski K., Malawska B. (2014). Antiallodynic and antihyperalgesic activity of 3-[4-(3-trifluoromethyl-phenyl)-piperazin-1-yl]-dihydrofuran-2-one compared to pregabalin in chemotherapy-induced neuropathic pain in mice. Pharmacol. Biochem. Behav..

[33] Gao W., Zan Y., Wang Z.J., Hu X.Y., Huang F. (2016). Quercetin ameliorates paclitaxel-induced neuropathic pain by stabilizing mast cells, and subsequently blocking PKCε-dependent activation of TRPV1. Acta Pharmacol. Sin..

[34] Singh J., Saha L., Singh N., Kumari P., Bhatia A., Chakrabarti A. (2019). Study of nuclear factor-2 erythroid related factor-2 activator, berberine, in paclitaxel induced peripheral neuropathy pain model in rats. J. Pharm. Pharmacol..

[35] Carvalho L.F., Silva A.M.F., Carvalho A.A. (2017). The use of antioxidant agents for chemotherapy-induced peripheral neuropathy treatment in animal models. Clin. Exp. Pharmacol. Physiol..

[36] Zhou Y.Q., Liu D.Q., Chen S.P., Chen N., Sun J., Wang X.M., Li D.Y., Tian Y.K., Ye D.W. (2020). PPARγ activation mitigates mechanical allodynia in paclitaxel-induced neuropathic pain via induction of Nrf2/HO-1 signaling pathway. Biomed. Pharmacother..

[37] Su C.J., Zhang J.T., Zhao F.L., Xu D.L., Pan J., Liu T. (2023). Resolvin D1/N-formyl peptide receptor 2 ameliorates paclitaxel-induced neuropathic pain through the activation of IL-10/Nrf2/HO-1 pathway in mice. Front. Immunol..

[38] Suárez-Rojas I., Pérez-Fernández M., Bai X., Martínez-Martel I., Intagliata S., Pittalà V., Salerno L., Pol O. (2023). The Inhibition of Neuropathic Pain Incited by Nerve Injury and Accompanying Mood Disorders by New Heme Oxygenase-1 Inducers: Mechanisms Implicated. Antioxidants.

[39] Toma W., Kyte S.L., Bagdas D., Alkhlaif Y., Alsharari S.D., Lichtman A.H., Chen Z.J., Del Fabbro E., Bigbee J.W., Gewirtz D.A. (2017). Effects of paclitaxel on the development of neuropathy and affective behaviors in the mouse. Neuropharmacology.

[40] Chaplan S.R., Bach F.W., Pogrel J.W., Chung J.M., Yaksh T.L. (1994). Quantitative assessment of tactile allodynia in the rat paw. J. Neurosci. Methods.

[41] Walf A.A., Frye C.A. (2007). The use of the elevated plus maze as an assay of anxiety-related behavior in rodents. Nat. Protoc..

[42] Steru L., Chermat R., Thierry B., Simon P. (1985). The tail suspension test: A new method for screening antidepressants in mice. Psychopharmacology.

[43] Porsolt R.D., Le Pichon M., Jalfre M. (1977). Depression: A new animal model sensitive to antidepressant treatments. Nature.

[44] Manavi M.A., Fathian Nasab M.H., Mohammad Jafari R., Dehpour A.R. (2023). Mechanisms underlying dose-limiting toxicities of conventional chemotherapeutic agents. J. Chemother..

[45] Flatters S.J., Bennett G.J. (2004). Ethosuximide reverses paclitaxel- and vincristine-induced painful peripheral neuropathy. Pain.

[46] Ward S.J., McAllister S.D., Kawamura R., Murase R., Neelakantan H., Walker E.A. (2014). Cannabidiol inhibits paclitaxel-induced neuropathic pain through 5-HT(1A) receptors without diminishing nervous system function or chemotherapy efficacy. Br. J. Pharmacol..

[47] Brandolini L., Benedetti E., Ruffini P.A., Russo R., Cristiano L., Antonosante A., d’Angelo M., Castelli V., Giordano A., Allegretti M. (2017). CXCR1/2 pathways in paclitaxel-induced neuropathic pain. Oncotarget.

[48] Galley H.F., McCormick B., Wilson K.L., Lowes D.A., Colvin L., Torsney C. (2017). Melatonin limits paclitaxel-induced mitochondrial dysfunction in vitro and protects against paclitaxel-induced neuropathic pain in the rat. J. Pineal Res..

[49] Miao H., Li R., Chen D., Hu J., Chen Y., Xu C., Wen Z. (2021). Pro-tective Effects of Vitamin E on Chemotherapy-Induced Peripheral Neuropathy: A Me-ta-Analysis of Randomized Controlled Trials. Ann. Nutr. Metab..

[50] Uher T., Bob P. (2013). Neuropathic pain, depressive symptoms, and C-reactive protein in sciatica patients. Int. J. Neurosci..

[51] Fidanboylu M., Griffiths L.A., Flatters S.J. (2011). Global inhibition of reactive oxygen species (ROS) inhibits paclitaxel-induced painful peripheral neuropathy. PLoS ONE.

[52] Coral-Pérez S., Martínez-Martel I., Martínez-Serrat M., Batallé G., Bai X., Leite-Panissi C.R.A., Pol O. (2022). Treatment with Hydrogen-Rich Water Improves the Nociceptive and Anxio-Depressive-like Behaviors Associated with Chronic Inflammatory Pain in Mice. Antioxidants.

[53] Ramos-Hryb A.B., Pazini F.L., Costa A.P., Cunha M.P., Kaster M.P., Rodrigues A.L.S. (2022). Role of heme oxygenase-1 in the antidepressant-like effect of ursolic acid in the tail suspension test. J. Pharm. Pharmacol..

[54] Bae E.H., Greenwald M.K., Schwartz A.G. (2021). Chemotherapy-Induced Peripheral Neuropathy: Mechanisms and Therapeutic Avenues. Neurotherapeutics.

[55] Duggett N.A., Griffiths L.A., McKenna O.E., de Santis V., Yongsanguanchai N., Mokori E.B., Flatters S.J. (2016). Oxidative stress in the development, maintenance and resolution of paclitaxel-induced painful neuropathy. Neuroscience.

[56] Shim H.S., Bae C., Wang J., Lee K.H., Hankerd K.M., Kim H.K., Chung J.M., La J.H. (2019). Peripheral and central oxidative stress in chemotherapy-induced neuropathic pain. Mol. Pain.

[57] Catanzaro E., Calcabrini C., Turrini E., Sestili P., Fimognari C. (2017). Nrf2: A potential therapeutic target for naturally occurring anticancer drugs?. Expert Opin. Ther. Targets.

[58] Raghunath A., Sundarraj K., Nagarajan R., Arfuso F., Bian J., Kumar A.P., Sethi G., Perumal E. (2018). Antioxidant response elements: Discovery, classes, regulation and potential applications. Redox Biol..

[59] Schipper H.M., Song W., Tavitian A., Cressatti M. (2019). The sinister face of heme oxygenase-1 in brain aging and disease. Prog. Neurobiol..

[60] Liu R., Yang J., Li Y., Xie J., Wang J. (2023). Heme oxygenase 1: The roles of both good and evil in neurodegenerative diseases. J. Neurochem..

[61] Shan Y., Lambrecht R.W., Donohue S.E., Bonkovsky H.L. (2006). Role of Bach1 and Nrf2 in up-regulation of the heme oxygenase-1 gene by cobalt protoporphyrin. FASEB J..

[62] Rosa P., Zerbinati C., Crestini A., Canudas A.M., Ragona G., Confaloni A., Iuliano L., Calogero A. (2018). Heme Oxygenase-1 and Brain Oxysterols Metabolism Are Linked to Egr-1 Expression in Aged Mice Cortex, but Not in Hippocampus. Front. Aging Neurosci..

[63] Pérez-Fernández M., Suárez-Rojas I., Bai X., Martínez-Martel I., Ciaffaglione V., Pittalà V., Salerno L., Pol O. (2023). Novel Heme Oxygenase-1 Inducers Palliate Inflammatory Pain and Emotional Disorders by Regulating NLRP3 Inflammasome and Activating the Antioxidant Pathway. Antioxidants.

[64] Neis V.B., Rosa P.B., Moretti M., Rodrigues A.L.S. (2018). Involvement of Heme Oxygenase-1 in Neuropsychiatric and Neurodegenerative Diseases. Curr. Pharm. Des..

[65] Majkutewicz I. (2022). Dimethyl fumarate: A review of preclinical efficacy in models of neurodegenerative diseases. Eur. J. Pharmacol..

[66] Shah S., Pushpa Tryphena K., Singh G., Kulkarni A., Pinjala P., Kumar Khatri D. (2024). Neuro-protective role of Carvacrol via Nrf2/HO-1/NLRP3 axis in Rotenone-induced PD mice model. Brain Res..

[67] Scibetta S., Miceli M., Iuliano M., Stefanuto L., Carbone E., Piscopo P., Petrozza V., Romeo G., Mangino G., Calogero A. (2024). In Vitro Evaluation of the Antioxidant Capacity of 3,3-Disubstituted-3H-benzofuran-2-one Derivatives in a Cellular Model of Neurodegeneration. Life.

[68] Zafar S., Luo Y., Zhang L., Li C.H., Khan A., Khan M.I., Shah K., Seo E.K., Wang F., Khan S. (2023). Daidzein attenuated paclitaxel-induced neuropathic pain via the down-regulation of TRPV1/P2Y and up-regulation of Nrf2/HO-1 signaling. Inflammopharmacology.

[69] Starobova H., Monteleone M., Adolphe C., Batoon L., Sandrock C.J., Tay B., Deuis J.R., Smith A.V., Mueller A., Nadar E.I. (2021). Vincristine-induced peripheral neuropathy is driven by canonical NLRP3 activation and IL-1β release. J. Exp. Med..

[70] Chen W., Wang X., Sun Q., Zhang Y., Liu J., Hu T., Wu W., Wei C., Liu M., Ding Y. (2022). The upregulation of NLRP3 inflammasome in dorsal root ganglion by ten-eleven translocation methylcytosine dioxygenase 2 (TET2) contributed to diabetic neuropathic pain in mice. J. Neuroinflamm..

[71] Silva Santos Ribeiro P., Willemen H.L.D.M., Versteeg S., Martin Gil C., Eijkelkamp N. (2023). NLRP3 inflammasome activation in sensory neurons promotes chronic inflammatory and osteoarthritis pain. Immunother. Adv..

[72] Chen C., Smith M.T. (2023). The NLRP3 inflammasome: Role in the pathobiology of chronic pain. Inflammopharmacology.

[73] Chen R., Yin C., Fang J., Liu B. (2021). The NLRP3 inflammasome: An emerging therapeutic target for chronic pain. J. Neuroinflamm..

[74] Tapia-Abellán A., Angosto-Bazarra D., Martínez-Banaclocha H., de Torre-Minguela C., Cerón-Carrasco J.P., Pérez-Sánchez H., Arostegui J.I., Pelegrin P. (2019). MCC950 closes the active conformation of NLRP3 to an inactive state. Nat. Chem. Biol..

[75] Mokhtari T., Uludag K. (2024). Role of NLRP3 Inflammasome in Post-Spinal-Cord-Injury Anxiety and Depression: Molecular Mechanisms and Therapeutic Implications. ACS Chem. Neurosci..

